# Identification of Predictive Factors for the Development of In-Transit Metastasis in Patients with Melanoma

**DOI:** 10.1245/s10434-025-17084-4

**Published:** 2025-03-10

**Authors:** Anne Huibers, Stanley P. Leong, Mohammed Kashani-Sabet, Richard L. White, John Vetto, Schlomo Schneebaum, Cristina O’Donoghue, Harrison Howard, Eli Avisar, Jukes P. Namm, Heidi Kosiorek, Mark Faries, Giorgos Karakousis, Jonathan S. Zager, Roger Olofsson Bagge, on behalf of the Sentinel Lymph Node Working Group

**Affiliations:** 1 Department of Surgery, Institute of Clinical Sciences, Sahlgrenska Academy, University of Gothenburg, Gothenburg, Sweden; 2 Department of Surgery, Sahlgrenska University Hospital, Gothenburg, Sweden; 3 Wallenberg Centre for Molecular and Translational Medicine, University of Gothenburg, Gothenburg, Sweden; 4 Center for Melanoma Research and Treatment, California Pacific Medical Center and Research Institute, San Francisco, CA USA; 5 Department of Surgery, Atrium Health Levine Cancer, Charlotte, NC USA; 6 Department of Surgery and Division of Surgical Oncology, Oregon Health and Science University, Portland, OR USA; 7 Department of Surgery, Tel Aviv University, Tel Aviv, Israel; 8 Department of Surgery, Rush University Medical Center, Chicago, IL USA; 9 Department of Surgery, University of South Alabama, Mobile, AL USA; 10 Department of Surgical Oncology, University of Miami School of Medicine, Miami, FL USA; 11 Department of Surgery, Loma Linda University, Loma Linda, CA USA; 12 Department of Quantitative Health Sciences, Mayo Clinic, Scottsdale, AZ USA; 13 Department of Surgery, Cedars-Sinai Medical Center, Angeles Clinic and Research Institute, Los Angeles, CA USA; 14 Division of Endocrine and Oncologic Surgery, University of Pennsylvania School of Medicine, Philadelphia, PA USA; 15 Departments of Cutaneous Oncology and Sarcoma, Moffit Cancer Center, Tampa, FL USA; 16 Department of Oncologic Sciences, University of South Florida Morsani College of Medicine, Tampa, FL USA

## Abstract

**Background:**

In patients with melanoma, in-transit metastasis (ITM) can develop. This study aimed to identify the risk for a first recurrence of ITM and associated predictive clinical factors in a large international cohort of patients with melanoma.

**Methods:**

Patients with primary cutaneous melanoma who underwent wide local excision (WLE) and sentinel lymph node biopsy (SLNB) were identified from the Sentinel Lymph Node Working Group (SLNWG) database between January 1993 and February 2023. Predictive factors for first recurrence of ITM were analyzed.

**Results:**

The study enrolled 7860 patients, and the median follow-up time was 47.1 months (interquartile range [IQR], 19.0–95.0 months). The risk for the development of ITM as a first recurrence was 4.12% (95% confidence interval [CI], 3.63–4.66%) at 5 years. The median time to first ITM recurrence was 15 months (IQR, 7.0–30.0 months). Significant clinicopathologic factors independently associated with an increased risk of ITM in multivariable analysis were increasing Breslow thickness (hazard ratio [HR], 1.37; 95% CI, 1.30–1.43; *p *< 0.0001), lower-extremity versus trunk melanoma (HR, 2.49; 95% CI, 1.86–3.32; *p *< 0.0001), increasing age (HR, 1.03; 95% CI, 1.02–1.04; *p *< 0.0001), number of positive sentinel lymph nodes (SLNs: 1 vs. 0 [HR, 2.24; 95% CI, 1.66–3.01; *p *< 0.0001] and 2 vs. 0 [HR, 2.37; 95% CI, 1.45–3.88; *p* = 0.0006]), and presence of vascular invasion (HR, 1.79; 95% CI, 1.21–2.64; *p* = 0.0035).

**Conclusion:**

The independent risk factors for the development of ITM identified in a large international cohort of melanoma patients were Breslow thickness, lower-extremity melanoma, older age, number of positive SLNs, and presence of vascular invasion.

The incidence of melanoma is increasing on a global scale, and in Western societies, approximately 1 in every 50 persons will experience melanoma.^1^ Melanoma can metastasize both via the lymphatics and through hematogenous dissemination, and a specific type of recurrence is in-transit metastases (ITMs). The ITMs appear as subcutaneous or cutaneous deposits of melanoma between the primary site and the regional nodal basin.

It has been reported that approximately 5–10% of patients with high-risk primary melanoma will experience ITM.^2,3^ In-transit metastasisis a heterogeneous disease, and the exact mechanism behind the development of ITM is not completely understood. However, evidence suggests that it originates from tumor cell emboli that become lodged within lymphatic vessels situated between the primary tumor site and nearby regional lymph nodes.^4^

The presence of ITM is a poor prognostic factor. In a large study of 11,614 patients treated at the Melanoma Institute of Australia between 1994 and 2009, the 5-year overall survival (OS) after diagnosis of ITM was 32.8%.^3^ However, this was largely before the introduction of modern systemic treatments for advanced melanoma. However, even with effective systemic treatments, ITM remains clinically challenging to treat, and evidence from prospective clinical trials on the use of immunotherapy is lacking.^5,6^

For patients with limited ITM, surgical resection usually is recommended. For patients with more extensive disease, regional therapies, such as intralesional therapies or isolated limb infusion/perfusion, are potential options.^7,8^

Interestingly, a subset of patients with ITM rapidly progress to distant metastasis, whereas others remain stable with regional disease for prolonged periods and can have long-term survival. This heterogeneity requires an individualized approach that aims for both locoregional control of disease and reduction of the risk for distant metastasis. Currently, we lack predictive factors to stratify patients at high risk for the development of ITM.

This study aimed to identify prognostic clinical and pathologic factors for the development of ITM in patients with melanoma undergoing a wide local excision (WLE) and SLNB in order to develop more individualized treatment and surveillance algorithms.

## Patients and methods

The study cohort included patients from the Sentinel Lymph Node Working Group (SLNWG) database between January 1993 and February 2023. Patients were eligible for this study if they were 18 years old or older, had a diagnosis of primary cutaneous melanoma with no evidence of satellite metastasis or ITM at diagnosis, and had undergone WLE and SLNB. Institutional review board approval was obtained by all the participating members, and data were collected through an encrypted, password-protected database.

The SLNWG is an initiative of the Sentinel Node Oncology Foundation and comprises 15 academic medical centers/practices in North America, Europe, and Asia. The SLNWG has assembled a comprehensive database of 12,143 patients with a diagnosis of stages I–IV melanoma between October 1993 and March 2024. The database includes demographic features, clinicopathologic characteristics, treatments, oncologic outcomes, and long-term follow-up evaluation.

Each participating center followed its national standards for diagnostic excision, wide local excision, sentinel lymph node biopsy (SLNB) and pathologic evaluation of the specimens, follow-up evaluation, and recording of the clinical outcomes. The members of SLNWG obtained ethical approval from their respective institutions for the use of the de-identified data for research and approval to share the data with the SLNWG database. Specific details concerning this can be shared on request from the corresponding author. Specimens were evaluated in accordance with the pathology guidelines specific to each participating institution, and no re-evaluation of pathologic specimens was undertaken.

The primary end point of the study was first recurrence of melanoma as ITM, defined as a skin or subcutaneous recurrence between the primary tumor and regional nodal basis (including both in-transit and satellite metastasis). Time to first ITM was defined as the time between the date of surgery (WLE + SLNB) and the date of ITM diagnosis. Within this time frame, patients could not have experienced a nodal recurrence or distant metastasis.

The following demographic and clinicopathologic variables were assessed: age, sex, melanoma type, region of primary melanoma, Breslow thickness, ulceration, Clark’s level of invasion, mitotic rate, pre-existing lesion, micro-satellitosis, primary tumor regression, perineural invasion, diagnostic radicality, diagnostic type, total number of sentinel lymph nodes (SLNs) removed, and total number of positive SLNs. Pre-existing lesion was defined as the presence of a pre-existing lesion at the site of the primary melanoma according to information in physician notes or on the pathology report. Diagnostic radicality was defined as the presence or absence of tumor cells in margins of the diagnostic excision.

Parametric continuous variables were described by mean and standard deviation, whereas non-parametric data were described as median and range or interquartile range (IQR), as applicable. Categorical variables were described by frequency and percentage. For comparisons between two groups, the following tests were used: Fisher’s exact test for dichotomous variables, chi-square test for non-ordered categorical variables, Mantel-Haenszel chi-square trend test for ordered categorical variables, and Mann-Whitney *U* test for continuous variables. Crude event rates were calculated as the number of events divided by the number of follow-up months per study group and expressed by 10,000 person-months. The 95% confidence interval for the event rate was estimated using exact Poisson limits. Time to ITM was described by unadjusted cumulative incidence curves handling death as a competing risk.

To estimate the risk for ITM given different baseline variables, Cox proportional hazards models were performed. Multivariable stepwise forward and backward regressions were applied to identify independent statistically significant predictors. Variables significant in the univariable analysis after Bonferroni-Holm adjustment were selected for potential qualification in a multivariable model. Interaction between different variables and non-linear effects of continuous variables were examined.

All tests were two-tailed. All analyses were performed using SAS software version 9.4 (SAS Institute Inc, Cary, NC, USA).

## Results

**Fig. 1:**
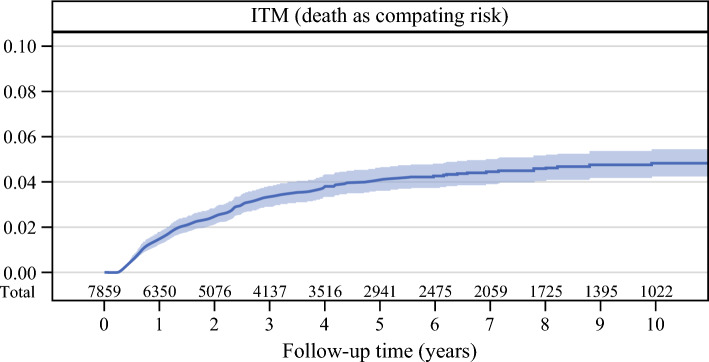
Risk of experiencing in-transit metastasis as first recurrence with death as a competing risk

The study included 7860 patients with a median follow-up period of 47.1 months (IQR, 19.0–95.0 months). The mean age was 58.7 ± 15.4 years, and 58% (4529/7860) of the patients were males. The rate of ITM development as a first recurrence was 4.12% (95% CI, 3.63–4.66%) at 5 years and 4.81% (95% CI, 4.23–5.44%) at 10 years, with a median interval from WLE and SLNB to first recurrence of 15 months (IQR, 7–30 months). Among the patients who experienced an ITM as a first recurrence, only 7% (18/264) experienced it longer than 5 years after the primary surgery, whereas 81% (213/264) of the patients experienced ITM within 3 years (Fig. 1). A comparison of the demographic and clinical characteristics between the patients with and without ITM is presented in Table 1.

**Table 1:** Baseline demographics and clinical characteristics for patients with and without in-transit metastasis (ITM)

	Total (*n* = 7860) *n* (%)	No ITM (*n* = 7596) *n* (%)	ITM (*n* = 264) *n* (%)	*p* Value
Microsatellitosis				0.035
No	4682 (59.6)	4522 (59.5)	160 (60.6)	
Yes	182 (2.3)	170 (2.2)	12 (4.5)	
Unknown	2996 (38.1)	2904 (38.2)	92 (34.8)	
Age (years) Range	58.7 ± 15.4 60 (18–98)	58.5 ± 15.4 60 (18–98)	64.0 ± 14.4 66 (18–92)	<0.0001
Age (quintiles)				<0.0001
≤45	1628 (20.7)	1600 (21.1)	28 (10.6)	
45 to <56	1653 (21.0)	1612 (21.2)	41 (15.5)	
56 to <64	1478 (18.8)	1429 (18.8)	49 (18.6)	
64 to <73	1650 (21.0)	1579 (20.8)	71 (26.9)	
>73	1451 (18.5)	1376 (18.1)	75 (28.4)	
Sex				0.34
Female	3331 (42.4)	3227 (42.5)	104 (39.4)	
Male	4529 (57.6)	4369 (57.5)	160 (60.6)	
Melanoma type				<0.0001
Superficial spreading	2206 (28.1)	2148 (28.3)	58 (22.0)	
Nodular	1421 (18.1)	1343 (17.7)	78 (29.5)	
Acrial lentignous	244 (3.1)	216 (2.8)	28 (10.6)	
Desmoplastic	173 (2.2)	167 (2.2)	6 (2.3)	
Other	1569 (20.0)	1531 (20.2)	38 (14.4)	
Unknown	2247 (28.6)	2191 (28.8)	56 (21.2)	
Primary region				<0.0001
Head & neck	1371 (17.5)	1336 (17.6)	35 (13.3)	
Upper extremity	1712 (21.8)	1675 (22.1)	37 (14.0)	
Lower extremity	1764 (22.5)	1650 (21.7)	114 (43.2)	
Trunk	3004 (38.3)	2926 (38.6)	78 (29.5)	
Missing	9	9	0	
Breslow thickness (mm) Mean ± SD Median (IQR)	2.4 ± 1.8 2 (0–10)	2.3 ± 1.8 2 (0–10)	3.7 ± 2.1 3 (1–10)	<0.0001
Breslow thickness (mm)				<0.0001
≤1.0	1670 (21.5)	1660 (22.1)	10 (3.8)	
1.1–2.0	2926 (37.6)	2866 (38.1)	60 (22.7)	
2.1–4.0	1815 (23.3)	1733 (23.0)	82 (31.1)	
>4.0	1373 (17.6)	1261 (16.8)	112 (42.4)	
Missing	76	76	0	
Ulceration				<0.0001
No	4922 (62.6)	4797 (63.2)	125 (47.3)	
Yes	2147 (27.3)	2025 (26.7)	122 (46.2)	
Unknown	791 (10.1)	774 (10.2)	17 (6.4)	
Clark´s level of invasion				<0.0001
I–III	1888 (24.0)	1847 (24.3)	41 (15.5)	
IV	3384 (43.1)	3253 (42.8)	131 (49.6)	
V	466 (5.9)	427 (5.6)	39 (14.8)	
Unknown	2122 (27.0)	2069 (27.2)	53 (20.1)	
Mitotic rate				0.016
<1/mm^2^	572 (7.3)	564 (7.4)	8 (3.0)	
>1/mm^2^	3695 (47.0)	3573 (47.0)	122 (46.2)	
Unknown	3593 (45.7)	3459 (45.5)	134 (50.8)	
Pre-existing nevus				<0.0001
Absent	1920 (24.4)	1838 (24.2)	82 (31.1)	
Present	2333 (29.7)	2291 (30.2)	42 (15.9)	
Unknown	3607 (45.9)	3467 (45.6)	140 (53.0)	
Regression status				0.0070
No	4275 (54.4)	4138 (54.5)	137 (51.9)	
Yes	944 (12.0)	925 (12.2)	19 (7.2)	
Unknown	2641 (33.6)	2533 (33.3)	108 (40.9)	
Vascular invasion				<0.0001
No	4999 (63.6)	4856 (63.9)	143 (54.2)	
Yes	419 (5.3)	386 (5.1)	33 (12.5)	
Unknown	2442 (31.1)	2354 (31.0)	88 (33.3)	
Perineural invasion				0.10
No	3366 (42.8)	3267 (43.0)	99 (37.5)	
Yes	168 (2.1)	159 (2.1)	9 (3.4)	
Unknown	4326 (55.0)	4170 (54.9)	156 (59.1)	
Diagnostic radicality				0.83
Negative	2895 (36.8)	2802 (36.9)	93 (35.2)	
Positive	2693 (34.3)	2602 (34.3)	91 (34.5)	
Unknown	2272 (28.9)	2192 (28.9)	80 (30.3)	
Diagnostic type				0.013
Excisional biopsy	2757 (35.1)	2655 (35.0)	102 (38.6)	
Incisional biopsy or punch biopsy	760 (9.7)	732 (9.6)	28 (10.6)	
Shave biopsy	1887 (24.0)	1846 (24.3)	41 (15.5)	
Unknown	2456 (31.2)	2363 (31.1)	93 (35.2)	
SLNB total no. of nodes				0.047
1	2671 (34.0)	2565 (33.8)	106 (40.2)	
2	2149 (27.3)	2081 (27.4)	68 (25.8)	
3	1238 (15.8)	1195 (15.7)	43 (16.3)	
>3	1587 (20.2)	1550 (20.4)	37 (14.0)	
Unknown	215 (2.7)	205 (2.7)	10 (3.8)	
SLND no. of positive nodes				<0.0001
0	6451 (82.1)	6279 (82.7)	172 (65.2)	
1	1002 (12.7)	936 (12.3)	66 (25.0)	
2	234 (3.0)	215 (2.8)	19 (7.2)	
>2	64 (0.8)	61 (0.8)	3 (1.1)	
Unknown	109 (1.4)	105 (1.4)	4 (1.5)	

SD, standard deviation; IQR, interquartile range; SLNB, sentinel lymph node biopsy; SLND, sentinel lymph node dissection

**Fig. 2:**
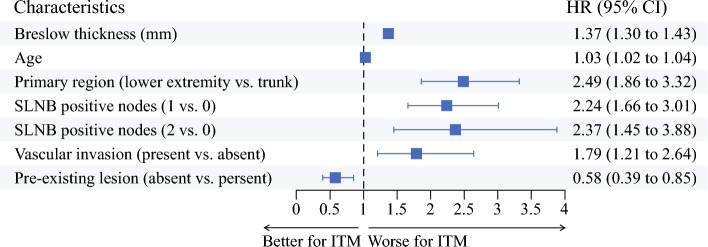
Forest plot showing hazard ratio (95% confidence interval; *p* value) for the independently associated risk factors of in-transit metastasis (ITM) recurrence

The patients who experienced ITM were older, with a mean age of 64.0 ± 14.4 years compared with 58.5 ± 15.4 years (*p* < 0.0001). The histologic subtype of melanoma varied significantly between the groups. Nodular melanoma was more prevalent in the ITM group (29.5% vs. 17.7%; *p *< 0.0001), whereas superficial spreading melanoma was less common (22.0% vs. 28.3%; *p *< 0.0001). The patients who experienced ITM as a first recurrence had tumors with significantly greater Breslow thickness (3.7 vs. 2.3 mm; *p *< 0.0001), had a higher Clark´s level of invasion (IV–V: 64.4% vs. 48.4%; *p *< 0.0001), were more often ulcerated (46.2% vs. 26.7%; *p *< 0.0001), more often had vascular invasion (12.5% vs. 5.1%; *p *< 0.0001), and as less often experienced a pre-existing lesion (31.1% vs. 24.4%; *p *< 0.0001) (Fig. 2).

The anatomic distributions of the primary melanoma differed significantly, with the lower extremity as the most common site in patients who experienced ITM (43.2%) and the trunk as the most common site in patients who did not (38.6%) (*p *< 0.0001). The patients who experienced ITM as a first recurrence included a significantly greater number of patients with a positive SLNB (*p *< 0.0001).

Of 7860 patients, 7776 had complete data available for a multivariable analysis. Both uni- and multivariable analyses are shown in Table 2. The clinical factors significantly associated with an increased risk of ITM in the univariable analysis were age, melanoma type, primary region, Breslow thickness, ulceration, Clark´s level of invasion, mitotic rate, absence of a pre-existing lesion, regression status, vascular invasion, diagnostic radicality, and SLNB positivity.

**Table 2:** Uni- and multivariable Cox proportional hazards models for time to in-transit metastasis (ITM)

Predictor	Value/comparison	Univariable analyses		Multivariable analyses	
		HR (95% CI)	*p* Value	HR (95% CI)	*p* Value
Age		1.03 (1.02–1.04)	<0.0001	1.03 (1.02–1.04)	<0.0001
Age (quintiles)	≤45 45<− 56 56<− 64 64<− 73 >73	Ref 0.67 (0.41–1.08) 1.42 (0.94–2.14) 1.89 (1.29–2.78) 2.42 (1.66–3.55)	0.10 0.10 0.0012 <0.0001		
Sex	Male vs female	1.18 (0.92–1.51)	0.19		
Melanoma type	Superficial spreading Acral lentiginous Nodular Other Unknown	Ref 5.07 (3.23–7.96) 2.41 (1.72–3.39) 1.00 (0.68–1.48) 0.91 (0.63–1.31)	<0.0001 <0.0001 0.99 0.59		
Primary region	Trunk Head & neck Lower extremity Upper extremity	Ref 1.15 (0.77–1.72) 2.56 (1.92–3.42) 0.87 (0.59–1.29)	0.48 <0.0001 0.50	1.10 (0.73–1.65) 2.49 (1.86–3.32) 0.85 (0.57–1.26)	0.64 <0.0001 0.42
Breslow thickness (mm)		1.37 (1.30–1.43)	<0.001	1.37 (1.30–1.43)	<0.001
Breslow groups	≤1.0 mm 1.1–2.0 mm 2.1–4.0 mm >4 mm	Ref 3.18 (1.63–6.21) 7.79 (4.04–15.02) 17.45 (9.13–33.34)	0.0007 <0.0001 <0.0001	2.75 (1.41–5.39) 5.44 (2.80–10.55) 10.74 (5.54–20.79)	0.0031 <0.0001 <0.0001
Ulceration	No Yes Unknown	Ref 2.63 (2.05–3.38) 0.71 (0.43–1.19)	<0.0001 0.19		
Clark´s level of invasion	I–III IV V Unknown	Ref 2.10 (1.48–2.99) 5.47 (3.53–8.50) 1.52 (1.01–2.28)	<0.0001 <0.0001 0.045		
Mitotic rate	<1/mm^2^ >1/mm^2^ Unknown	Ref 2.41 (1.18–4.93) 2.13 (1.05–4.35)	0.016 0.037		
Microsatellitosis	No Yes Unknown	Ref 2.32 (1.29–4.17) 0.87 (0.67–1.13)			
Pre-existing lesion	Absent Present Unknown	Ref 0.40 (0.28–0.58) 0.76 (0.58–1.00)	<0.0001 0.052	0.58 (0.39–0.85) 0.88 (0.65–1.19)	0.0056 0.40
Regression status	No Yes Unknown	Ref 0.59 (0.36–0.95) 1.14 (0.89–1.47)	0.029 0.30		
Vascular invasion	No Yes Unknown	Ref 3.02 (2.07–4.41) 1.15 (0.88–1.50)	<0.0001 0.30	1.79 (1.21–2.64) 1.28 (0.95–1.72)	0.0035 0.10
Perineural invasion	No Yes Unknown	1.79 (0.90–3.54) 0.90 (0.70–1.16)	0.09 0.42		
Diagnostic radicality	Negative Positive Unknown	Ref 1.30 (0.97–1.73) 0.98 (0.73–1.32)	0.08 0.89		
Diagnostic type	Excisional biopsy Incisional or punch biopsy Shave biopsy Unknown	Ref 1.13 (0.74–1.72) 0.76 (0.53–1.09) 0.93 (0.70–1.23)	0.56 0.13 0.62		
SLNB total nodes	1 2 3 >3 Unknown	Ref 0.78 (0.58–1.06) 0.83 (0.59–1.19) 0.58 (0.40–0.84) 1.58 (0.82–3.02)	0.11 0.31 0.0038 0.17		
SLNB positive nodes	0 1 2 >2 Unknown	Ref 3.21 (2.42–4.27) 3.99 (2.48–6.42) 2.52 (0.80–7.89) 2.00 (0.74–5.40)	<0.0001 <0.0001 0.11 0.17	2.24 (1.66–3.01) 2.37 (1.45–3.88) 1.32 (0.42–4.15) 2.33 (0.86–6.32)	<0.0001 0.0006 0.64 0.10

HR, hazard ratio; CI, confidence interval; SLNB, sentinel lymph node biopsy

The significant variables in the univariable analysis, with a *p* value lower than 0.05, were included in a stepwise multivariable Cox regression analysis. This resulted in the following significant and independently associated risk factors associated with an increased risk of ITM as a first recurrence: increasing Breslow thickness (HR, 1.37; 95% CI, 1.30–1.43; *p *< 0.0001), lower-extremity versus trunk melanoma (HR, 2.49; 95% CI, 1.86–3.32; *p *< 0.0001), older age (HR, 1.03; 95% CI, 1.02–1.04; *p *< 0.0001), number of positive SLNs (1 vs. 0 [HR, 2.24; 95% CI, 1.66–3.01; *p *< 0.0001] and 2 vs. 0 [HR, 2.37; 95% CI, 1.45–3.88; *p* = 0.0006]), and presence of vascular invasion (HR, 1.79; 95% CI, 1.21–2.64; *p* = 0.0035). However, the presence of a nevus-associated primary melanoma (pre-existing nevus) was protective against ITM recurrence (HR, 0.58; 95% CI, 0.39–0.85; *p* = 0.0056).

## Discussion

In this large multi-institutional and international retrospective registry study, we found the 5-year risk for development of an ITM as a first recurrence to be 4.12% and the 10-year risk to be 4.81%. A majority (81%) of all the patients who experienced an ITM recurrence did so within 3 years, and the risk of experiencing an ITM after 5 years was very low. Breslow thickness, lower-extremity melanoma, age, number of positive SLNs, and vascular invasion all were strongly associated with the risk of ITM recurrence. Interestingly, in contrast to de novo melanoma, the presence of a pre-existing lesion appeared to be an independent protective factor against the development of ITM.^9^ Ulceration was significantly associated with ITM in the univariable analysis, but was no longer statistically significant in the multivariable analysis.

Our reported ITM risk rate of 4.81% was closely aligned with the 4.3% found in the large study by Read et al.^3^ from the MIA study (*n* = 11,614), and slightly higher than the 3.2% reported in the smaller study by Jakub et al.^10^ from the Mayo clinic (*n* = 854). The differences can likely be attributed to variations in study populations and follow-up times because our study had a longer median follow-up period of 47.1 months compared with 40.6 months in the MIA study and a duration more comparable with the 49.2 months reported in the Mayo study. These findings collectively support a consistent risk range for ITM across diverse cohorts.

In the MIA study, ITM was defined as cutaneous, intradermal, and subcutaneous metastases occurring 5 cm or further from the primary site, whereas it was defined as further than 2 cm in the Mayo study. The agreed upon definition of ITM in the National Comprehensive Cancer Network (NCCN) guidelines is a metastasis further than 2 cm beyond the primary tumor.^11^ The differentiation between satellite metastases and ITM in melanoma has traditionally been made on the basis of their presumed biologic behavior. However, there are compelling reasons to see them as one entity, and current guidelines suggest a combined definition that includes both ITM and satellite metastases as a locoregional recurrence.^12,13^

Several pathophysiologic mechanisms have been suggested for the development of in-transit recurrence, such as tumor cell dispersion through tissue fluid and implantation of tumor cells in the subcutaneous tissue after spread via the bloodstream.^14,15^ The hypothesis that it originates from tumor cell emboli entrapped in the dermal lymphatic vessels between the primary site and the regional lymph node basin is the most widely accepted.^3^ This suggests an analogous tumor mechanism and dissemination for both satellite metastasis and ITM. In clinical practice, our approach to managing locoregional recurrences, both satellite metastasis and ITM, is guided by the extent of the tumor burden, the interval since the last recurrence, individual patient characteristics, and available treatment options, but very seldom by distance from the primary tumor.

Our findings that SLN positivity was predictive for ITM further support the hypothesis of lymphatic spread as the pathophysiologic mechanism of ITM development. The association between a positive SLN and a higher incidence of ITM further supports the hypothesis of lymphatic spread, in which melanoma cells traverse lymphatic vessels to reach the SLN. Conversely, the significantly lower incidence of ITM in cases with a negative SLN indicates that melanoma cells typically remain confined to the primary site in the absence of SLN involvement. With an average size of up to 20 μm, cancer cells can easily enter lymphatic vessels, which have an average diameter of 100 μm, and travel toward the lymph nodes.^16^ It has been proposed that clusters of cancer cells may obstruct lymphatic vessel valves, potentially forming colonies along the vessel walls. These colonies could then invade surrounding tissues, contributing to ITM. Additionally, cancer cells may possess specific receptors enabling adhesion to the lymphatic endothelium, providing a mechanism for further growth and dissemination.^17^

In our study, we found that the median time to development of an ITM recurrence was 15 months after definitive resection of the primary melanoma, and that the risk for the development of an ITM after 5 years was low. Approximately 80% of all first recurrences of ITM were experienced within 3 years. Previous studies investigating the time to development of an ITM recurrence have reported similar recurrence intervals. The median time was reported to be 18 months in the aforementioned MIA study^3^ and 16 months in the study from the Mayo clinics.^10^ The low risk for the development of ITM beyond 5 years may reflect an incubation period, with ITM peaking approximately 3 years after treatment and declining thereafter. This pattern could be influenced by a dormancy phase of disseminated melanoma cells and the evolving role of the host immune system over time.

Our study identified a subset of clinicopathologic factors that were independently predictors of ITM recurrence: Breslow thickness, lower-extremity location, age, number of positive SLNs, and vascular invasion. These findings all align with the existing literature. Pawlik et al.^2^ reported on 91 patients who experienced ITMs in a cohort of nearly 1400 melanoma patients undergoing SLNB. Their analysis identified Breslow thickness, age older than 50, ulceration, and lower-extremity primary location as poor prognostic factors for ITM development. The current data could be used and validated in other datasets to identify a low-risk group of patients at risk for ITM.

A limitation of our study was the lack of routinely collected data on systemic treatments administered to patients with ITM, as well as data on nodal recurrence, distant metastasis-free survival, and overall survival.

In conclusion, in this large international cohort of patients with melanoma, the 5-year risk for the development of ITM as a first recurrence was 4.12%. The independent risk factors for the development of ITM were Breslow thickness, lower-extremity melanoma versus trunk melanoma, increasing age, number of positive SLNs, and presence of vascular invasion, whereas the presence of a pre-existing lesion in the primary tumor was a protective factor.
